# Bone Reconstruction Using Two-Layer Porcine-Derived Bone Scaffold Composed of Cortical and Cancellous Bones in a Rabbit Calvarial Defect Model

**DOI:** 10.3390/ijms23052647

**Published:** 2022-02-28

**Authors:** Yong-Ho Seo, Su-Hyun Hwang, Yu-Na Kim, Hyung-Joon Kim, Eun-Bin Bae, Jung-Bo Huh

**Affiliations:** 1 Department of Prosthodontics, Dental and Life Sciences Institute, Education and Research Team for Life Science on Dentistry, School of Dentistry, Pusan National University, Yangsan 50612, Korea; cigaronne@hanmail.net (Y.-H.S.); hsh2942@hanmail.net (S.-H.H.); 2 Department of Oral Physiology, Periodontal Diseases Signaling Network Research Center, Dental and Life Science Institute, School of Dentistry, Pusan National University, Yangsan 50612, Korea; kyn0394@naver.com (Y.-N.K.); hjoonkim@pusan.ac.kr (H.-J.K.); 3 Section of Restorative Dentistry, University of California, Los Angeles, CA 90095, USA

**Keywords:** bone regeneration, bone scaffold, cortical bone, cancellous bone, porcine, xenograft

## Abstract

In this study, we aimed to investigate the bone regeneration efficiency of two-layer porcine-derived bone scaffolds composed of cancellous and cortical bones in a rabbit calvarial defect model. Four circular calvaria defects were formed on cranium of rabbit and were filled with block bone scaffolds of each group: cortical bone block (Cortical group), cancellous bone block (Cancellous group), and two-layer bone block (2layer group). After 8 weeks, new bones were primarily observed in cancellous parts of the Cancellous and 2layer groups, while the Cortical group exhibited few new bones. In the results of new bone volume and area analyses, the Cancellous group showed the highest value, followed by the 2layer group, and were significantly higher than the Cortical group. Within the limitations of this study, the cancellous and two-layer porcine-derived bone scaffolds showed satisfactory bone regeneration efficiency; further studies on regulating the ratio of cortical and cancellous bones in two-layer bones are needed.

## 1. Introduction

A sufficient volume of residual bone is required for successful implant surgery [1]. A narrow bone width in the anterior or premolar resigns would adversely affect ideal implant placement. In particular, alveolar bones with moderate to severe periodontitis or bone defects caused by cysts or tumors require extensive bone grafting. Ideal bone substitutes should have no immune response and sufficient stem or progenitor cells to induce bone formation. Additionally, bone graft substitutes must be able to maintain their stability in bony defects and could promote rapid new bone formation and revascularization [2,3,4]. Although autologous bone grafts are considered the gold standard for bone regeneration procedures in that most conditions are met, they need additional surgery, and the bone amount that can be harvested is limited [5,6].

Among the various bone graft substitutes currently in use, xenografts are the most widely used along with synthetic bone substitutes [7]. Initially, the use of xenografts was restricted owing to the risk of autoimmune diseases, but their use has gradually been increasing with the development of protein separation technology [8]. In addition, xenografts have no limit on the usable bone mass and have characteristics similar to human bone, yielding superior osteogenesis performance to synthetic substitutes [9,10]. Even though bovine-derived xenografts have occupied the current bone material market, porcine-derived xenografts have also been continuously developed and showed sufficient results in bone regeneration compared to bovine xenografts [11,12,13]. Braceye et al. [13] reported that porcine bones exhibited cancellous bone structure, porosity, and microstructure of crystallites similar to human bones. Salamanca et al. [11] revealed that the chemical composition of porcine bone is approximated to that of human cancellous bone, which promotes internal growth of blood vessels and osteogenic cells and consequently increases bone formation. Porcine bone strength and Ca/P ratio are also more similar to human cancellous bone than bovine bone, and the recently developed porcine bone has a low concern of zoonosis; hence, it has sufficient commercial value in the bone regeneration field [14,15,16].

Most xenografts are in the form of particles with various sizes [17], and the particle type is usually used with the conventional method covering the membrane after compacting the bone substitutes into the defect [18]. When the defect shape is irregular or wide, it is difficult to expect the grafted particle to maintain stability during the reconstruction period [19]. When the stability of the graft substitutes is insufficient, it is difficult to achieve proper bone formation, which can lead to implant failure. Additionally, particle types lack bonding strength and thus have poor operability that may lead to the graft substitute being filled in the wrong position or being lost during filling [20,21]. The use of xenogeneic block bone grafts for the treatment of atrophic areas has emerged to overcome the problems associated with particle type. In line with Simon et al. [22], block bone was easy to manipulate and allowed for large amounts of new bone, which is beneficial for extensive bone loss. Gehrke et al. [23] demonstrated that xenograft block bone has an advantage for vital osteogenic cells because its internal trabecular bone structure and porosity are similar to those of humans. Block bone grafts with these characteristics can be an adequate alternative to supplement particle type.

Cortical and cancellous bones show different healing patterns during bone grafting [24]. Cortical bone, a hard outer layer of bone, is denser and has a smaller surface area than cancellous bone, which delays revascularization and reduces new bone formation [23]. Compared to cancellous bone, cortical bone has superior physical properties and distinguished structural stability in the early stage of surgery, being able to bear the load [25]. Cancellous bone consists of a network of trabecula and has a porous structure and a wide surface, so that the initial revascularization for new bone formation is rapid. In addition, cancellous bone is considered a better bone substitute than cortical bone because it can also gain enough mechanical strength after completing bone formation [26,27].

Nevertheless, there have been few reports reconstructing bony defects using a por-cine-derived block bone scaffold that maintains the existing bone structure composed of cortical and cancellous bone, other than using particle-type bone grafts [13]. If the characteristics of cancellous bone and cortical bone can be conferred to a single scaffold with two layers, high new bone formation performance of cancellous bone and ideal strength of cortical bone can be expected. Therefore, in this study, we aimed to investigate the bone regeneration efficiency of two-layer porcine bone scaffolds composed of cancellous and cortical bones compared to cortical bone and cancellous bone in the rabbit calvaria defect model.

## 2. Results

### 2.1. In Vitro Findings

#### 2.1.1. Scanning Electron Microscope (SEM) Surface Observation

**Figure 1:**
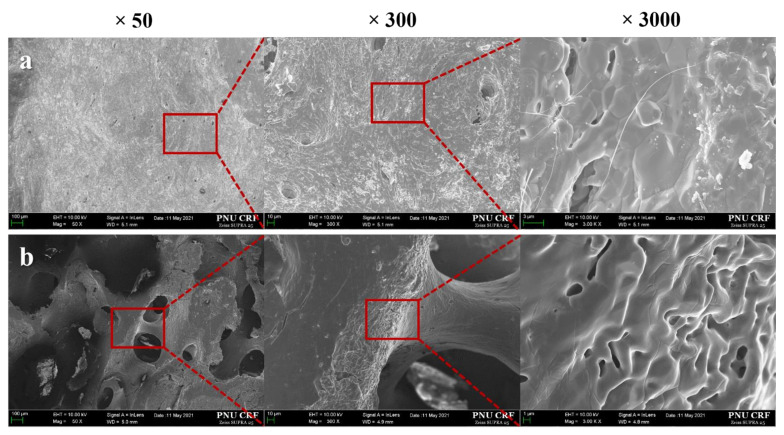
Scanning electron microscopy images of the surface. (**a**) Cortical group, (**b**) Cancellous group.

SEM micrographs of cortical and cancellous bone parts are presented in Figure 1. At the magnifications of ×50 and ×300, the surface morphologies of cancellous bone exhibited interconnective porous structure, while few pores were observed in the cortical bone. Larger micro-pores were observed in the cancellous bone surface compared to the cortical bone at the magnification of ×3000.

#### 2.1.2. Energy-Dispersive X-ray Spectroscopy (EDS) Findings

The results of the surface composition are summarized in Table 1. EDS showed Ca and P, which are major elements of the xenogeneic bone scaffold; C and O, which are major elements of the organic bone matrix; and Mg and Na as osteocyte differentiation elements. The ratios of Ca/P in both groups were 1.96% and 2.11%, respectively.

#### 2.1.3. Compressive Strength Analysis

**Figure 2:**
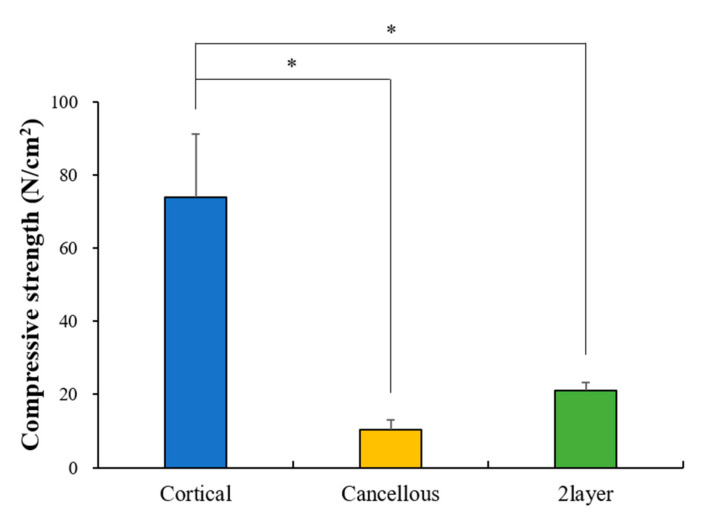
Compressive strength result (* *p* < 0.05; *n* = 10).

The results of compressive strength measurements (N/cm^2^) are shown in Figure 2. The Cortical group (74.01 ± 17.13) was significantly higher than other groups (*p* > 0.05). There was no significant difference between the Cancellous (10.39 ± 2.75) and 2layer groups (21.13 ± 2.27) (*p* < 0.05).

#### 2.1.4. Porosity Analysis

**Figure 3:**
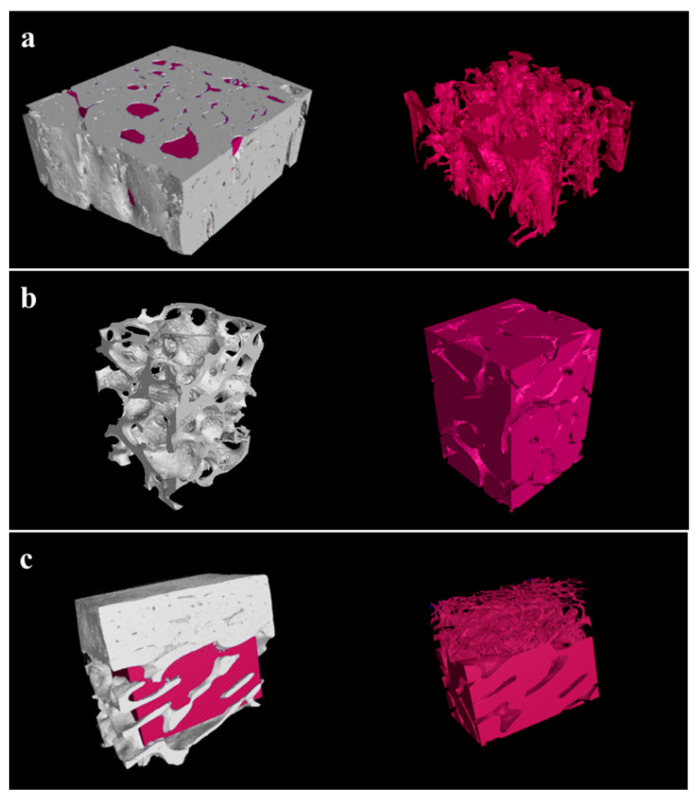
Three-dimensional porosity images of experimental groups. (**a**) Cortical, (**b**) Cancellous, and (**c**) 2layer bone scaffolds.

Macro-porosities of the Cortical, Cancellous, and 2layer groups were 19.45%, 83.21%, and 42.56%, respectively (Figure 3). The Cancellous group containing more macropores indicated the highest porosity compared to the Cortical group (Figure 3a,b). The 3D image of the 2layer group, which is composed of cortical and cancellous bones, shows two distinct layers with different porosities (Figure 3c).

#### 2.1.5. CCK-8 Assays of Cell Viability and Proliferation

**Figure 4:**
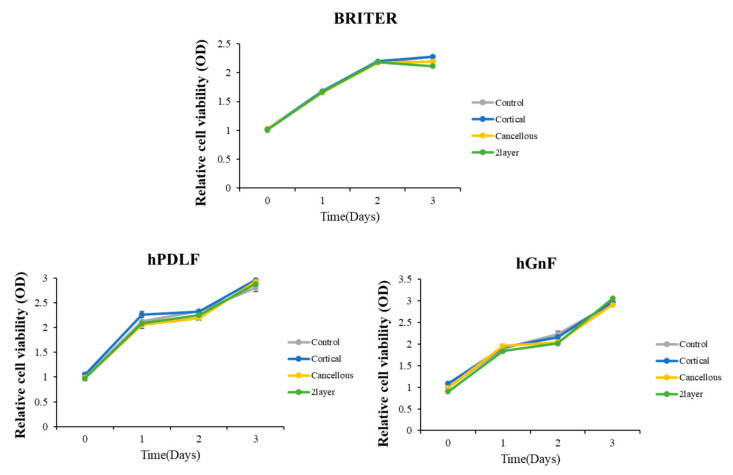
Cell viabilities of each group on mouse BMP responsive reporter osteoblast cell line (BRITER), human periodontal ligament fibroblasts (hPDLF), and human gingival fibroblasts (hGnF) cells (*p* > 0.05).

To analyze the possible cytotoxic effects of each bone block substitute, cell viabilities of BRRITER, hPDLF, and hGnF were measured by CCK-8 at 0, 1, 2, and 3 days after the cells were seeded and treated with basal culture media and three each bone block substitute extract solution media (Figure 4). The control and three extract solutions were treated with free media and each extract solution was diluted 20% in culture media and the results of cell viability according to absorbance by time point were normalized by the control (Figure 4). The cell morphology and number of cells observed between the control and diluted extract-treated groups were not significantly different during cultures of 1, 2, and 3 days. Moreover, cell viability measured by CCK-8 was not confirmed to be a significant difference between the control and three extracted solutions. These results suggest that three bone block substitutes did not effect on BRITER, hPDLF, and hGnF cell viability.

#### 2.1.6. Osteoblast Differentiation

**Figure 5:**
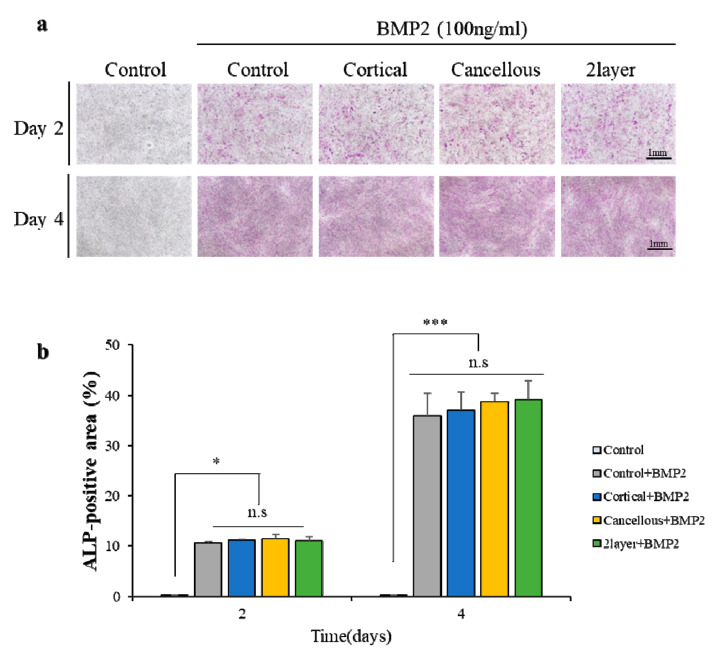
Pre osteoblast cells differentiation. (**a**) Alkaline phosphatase (ALP) staining of pre-osteoblastic BRITER cells in osteogenic media with BMP2 that diluted each bone block substitute extract solutions and (**b**) quantitative analysis (n.s represents no significance, * *p* < 0.05, *** *p* < 0.001).

To examine whether the three bone block substitute extracts could induce the osteoblast differentiation, the BRITER cell line induced the osteoblast differentiation through BMP2 (Figure 5). The differentiation was for 2 and 4 days, and the differentiation difference was confirmed by alkaline phosphatase (ALP) staining (Figure 5a) and quantitative analysis of the ALP-positive area (Figure 5b) of the BMP2 untreated control, the only BMP2-treated control, and the BMP2-treated diluted three bone block substitute extracts. On differentiation day 2, there was no difference in differentiation between four groups. In addition, on differentiation day 4, only the differentiation was progressed compared to day 2, but there was no significant difference between the BMP2-treated control group and the BMP2-treated diluted three bone block substitute extracts. These results suggest that the three bone block substitutes have no effects on the regulation of osteogenic differentiation in BRITER cells.

#### 2.1.7. Osteoblast Differentiation Marker Expression

**Figure 6:**
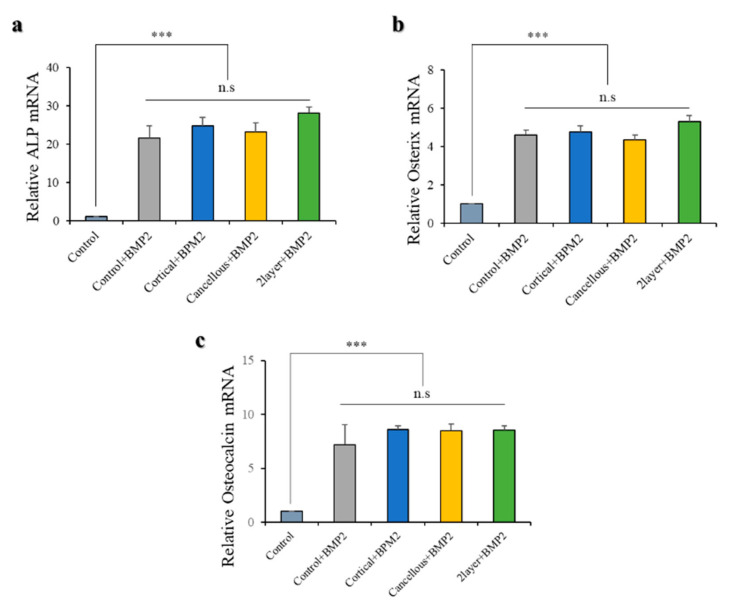
Quantitative real-time polymerase chain reaction (PCR) analysis of BRITER cells on diluted each bone block substitute extract solution media. Osteoblast differentiation was confirmed by qPCR analysis by osteoblast differentiation marker genes (**a**) *ALP*, (**b**) *Osterix*, and (**c**) *Osteocalcin*. *ALP, Osterix,* and *Osteocalcin* normalized to *β-actin* (n.s represents no significance, *** *p* < 0.001).

All the bone blocks had an equal effect on osteogenic differentiation. The expression of early markers of osteoblastic lineage—*ALP, Osterix, and Osteocalcin*—was confirmed by quantitative real-time PCR. Marker expression was confirmed in day 4 differentiated BRITER cells under the same conditions as Figure 5 (Figure 6). The marker expression between the only BMP2-treated control and the BMP2-treated diluted three bone block substitute extracts were compared. A significant difference was not confirmed in the three markers (Figure 6), which suggests that the three bone block substitutes have no effects on the regulation of osteogenic differentiation marker expression.

### 2.2. In Vivo Findings

#### 2.2.1. Clinical Findings

After the bone grafting in the rabbit calvaria defects, there was no inflammatory response or adverse tissue reaction. No signs of toxicity were observed during the experimental period.

#### 2.2.2. Volumetric Findings

**Figure 7:**
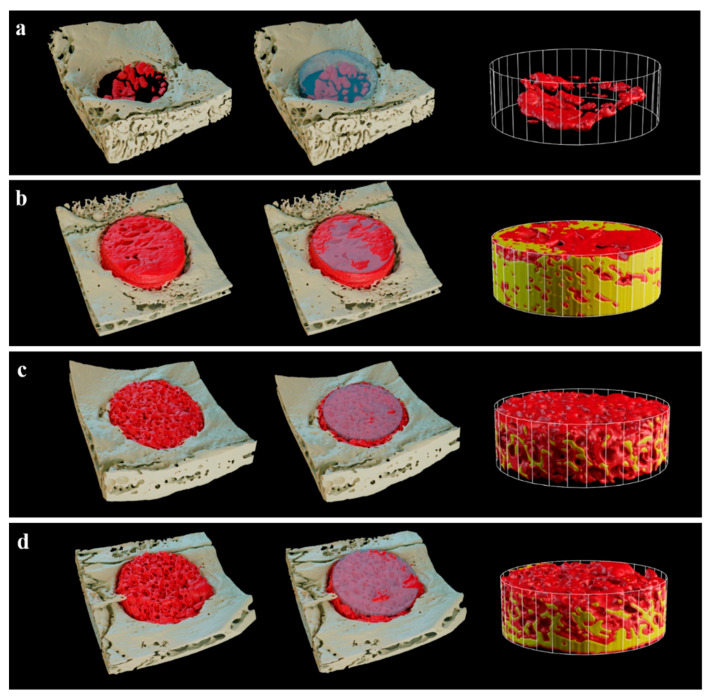
Three-dimensional micro CT images after 8 weeks. (**a**) Control, (**b**) Cortical, (**c**) Cancellous, and (**d**) 2layer groups. Red area: new bone, yellow area: grafted bone block.

In the 3D images of micro CT, the defects of the Cancellous and 2layer groups were filled with newly generated bones compared to the Control and Cortical groups (Figure 7). Notably, new bones in the Cancellous group exhibited even distribution within the ROI (Figure 7c). 

**Figure 8:**
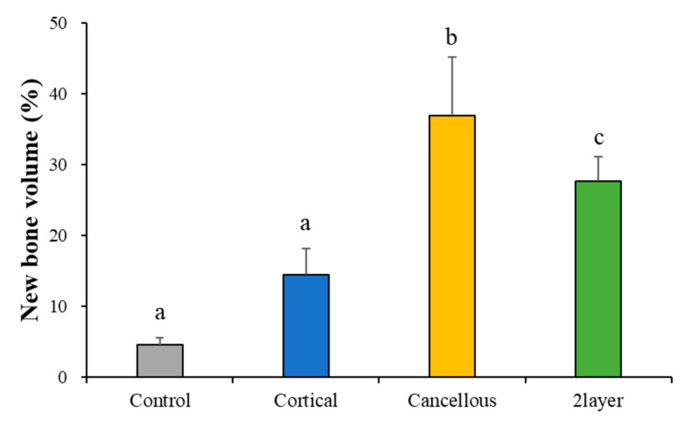
Volumetric measurement of newly formed bones. Different letters denote significant differences among each group (*p* < 0.001).

The volumetric results analyzed using µCT are summarized in Table 2 and Figure 8. The new bone volumes (%) of the Control, Cortical, Cancellous, and 2layer groups were 4.58 (±1.06), 14.45 (±3.72), 37.02 (±8.20), and 27.67 (±3.50), respectively. The Cancellous and 2layer groups showed significant differences from the Control. There was no significant difference between the Control and Cortical groups (*p* > 0.05).

#### 2.2.3. Histologic Findings

**Figure 9:**
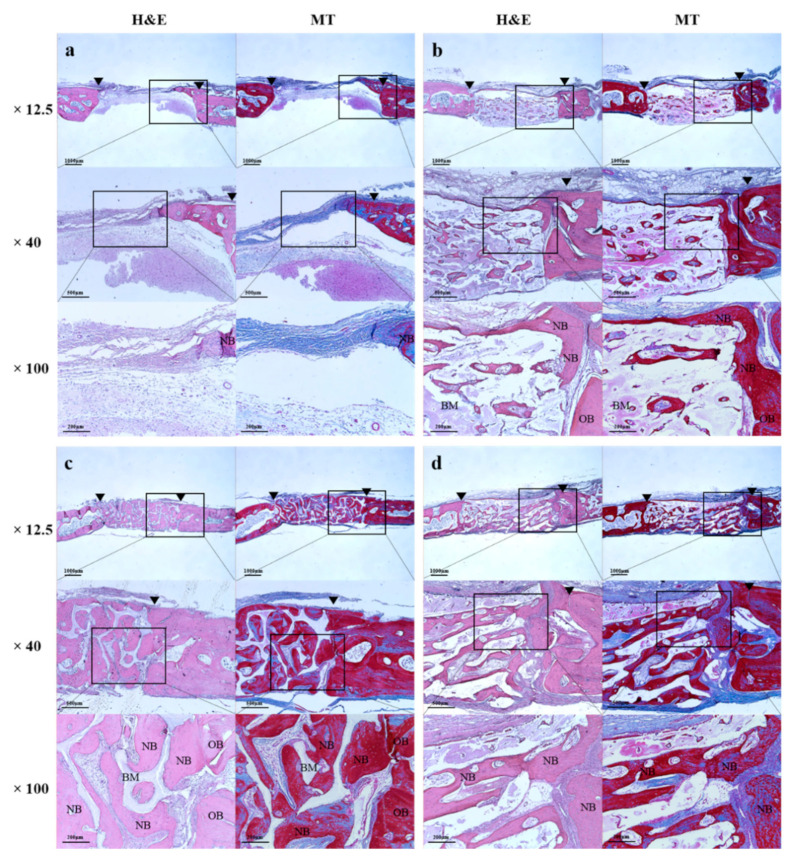
Histological images of experimental groups after 8 weeks. (**a**) Control, (**b**) Cortical, (**c**) Cancellous, and (**d**) 2layer groups. H&E: hematoxylin eosin staining, MT: Masson’s trichrome staining, OB: old bone, NB: newly formed bone, BM: bone graft material, arrow head: defect margin.

At 8 weeks, in all histological specimens, the grafted bone scaffolds were suitably located in the defects (Figure 9). In the Cortical, Cancellous, and 2layer groups, newly formed bones were generated into the pores (Figure 9b–d), and the new bone formation was more clearly observed in the cancellous area of the Cancellous and 2layer groups having large pores (Figure 9c,d). The defect of the Control remained and filled with fibrotic tissues without new bone formation (Figure 9a). In the cancellous part of the Cancellous and 2layer groups, osteoblasts were frequently detected surrounding the new bones and were denser than in the Control and Cortical groups. 

#### 2.2.4. Histometric Findings

**Figure 10:**
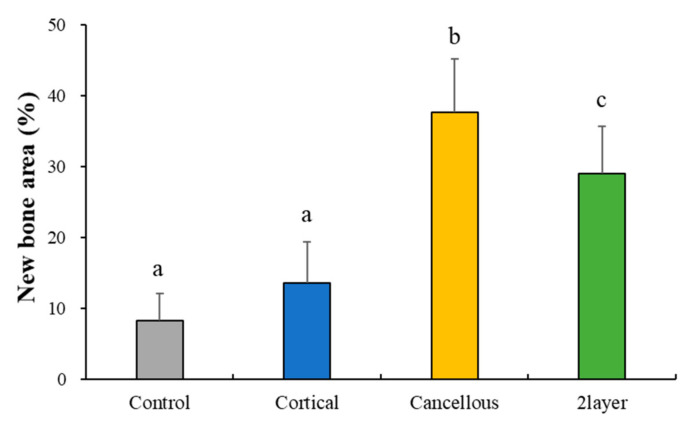
Histometric measurement of newly formed bones. Different letters denote significant differences among each group (*p* < 0.05).

Mean and standard deviations of the new bone area are shown in Table 3 and Figure 10. At 8 weeks, the new bone area (%) of the Control, Cortical, Cancellous, and 2layer groups were 8.37 (±3.77), 13.62 (±5.86), 37.76 (±7.44), and 29.12 (±6.61), respectively. The Cancellous and 2layer groups showed significant differences from the Control (*p* > 0.05). There was no significant difference between the Control and Cortical groups (*p* < 0.05).

## 3. Discussion

Xenografts can generate large amounts of bone harvest and have sufficient strength and a porous structure that actively drives cell migration, differentiation, and vascularization for new bone formation [28,29,30]. Among the types of xenografts, porcine-derived bones have been used for bone reconstruction because they have physiochemical characteristics similar to human bone [11,13]. Most bone graft products are in the form of particles, but in the case of bony defects that require a significant reconstruction, block bones are preferred over particle types to achieve enough stability during the bone-forming period [31]. Block bones are commonly formed by mixing bone particles with hydrogels, so they are not able to take or mimic the advantages of their own unique structure of cortical and cancellous bones [18,25,26,27]. Therefore, in the present study, we prepared a two-layer porcine-derived bone scaffold consisting of cortical and cancellous bones and compared the in vitro and in vivo bone regeneration efficiency to cortical and cancellous bone scaffolds.

The pore size and structure of bone graft materials play important roles in the migration and attachment of osteoblasts. According to Amid et al. [32], over 300 µm of micro-pores appear in human trabecular bones as well as in xenografts derived from bovine and porcine, and the connections between each pore are well formed. As reported by Bruzauskaite et al. [33], osteoblasts were optimally attached at a nanometric pore of 0.2–1 µm, and proliferation and differentiation occurred at a pore size of 5–8 µm. Hence, porous structures with various sizes of pores are mandatory in an ideal bone substitute. It has been reported that porcine-derived xenografts are similar to human bone with porous structures of various sizes, advantageous for forming new bones [34]. In this surface morphology observation using SEM, the cancellous bone scaffold exhibited both macro- and micro-pores. In contrast, most of the cortical bone scaffold was composed of a few micro-pores.

Porous structures of bone scaffolds promote not only the exchange of oxygen, waste products, and nutrients but also enable the maintenance and concentration of proteins that regulate differentiation and dedifferentiation [35]. Adachi et al. [36] reported that the larger the hole size was, the greater were the penetration and differentiation of osteoblasts helping bone regeneration. Cortical bones are compact and composed of a thin cylindrical structure called osteo, while cancellous bone encloses large interconnective pores that give a honeycomb structure allowing oncoming blood vessels and bone marrow [37]. As a result of these structural differences, in this three-dimensional macro-porosity analysis, the Cancellous group (83.21%) appeared to have the most porous structures connected to each other compared to the Cortical group (19.45%). Most of the porosity in the 2layer group (42.56%) is presented in the cancellous part. This high porosity seems to contribute to new bone formation ability.

Another important factor of block bone substitutes is structural stability and mechanical strength [38]. For structural stability, appropriate compressive strength is required. In this experiment, the Cortical group showed higher compressive strength than the Cancellous and 2layer groups. Suitable compressive strength can prevent deformation and structural collapse of graft material and is considered to be easy to process for appropriate size and shape at the transplant site. In addition, as a consequence of the membrane function of cortical bones, it can act as a defense mechanism suitable for the penetration of epithelialization by surrounding the epithelium, which may have the advantage of not requiring an additional membrane. However, despite the fact that the cortical bones showed the highest strength as a bone graft material, they are too dense for vascular regeneration, which might reduce bone regeneration [23].

Fibroblasts are known as mesenchymal cells that reside in mesenchymal stroma. In general, fibroblasts are involved in the formation of cell microstructures and regulate immunity and inflammatory responses in the recovery of damaged tissues [39,40]. They also exist in various tissues of the human body and have outstanding plasticity, and their differentiation ability is not much distinguished from MSC [41]. They can be differentiated in various ways depending on the experimental conditions and, in particular, in osteogenic cells. Therefore, the osteogenic characteristics of fibroblasts, which produce osteoblasts, are suitable for studying bone disease models or regenerative applications [42,43,44]. In this study, the biological efficacy and osteogenicity of each of the three xenogeneic bone substitutes were evaluated using fibroblasts derived from gingiva, the periodontal ligament surrounding the teeth, and using the osteoblast cell line. Cell proliferation data indicated that the three xenogeneic bone substitutes did not induce cell death during cell culture, meaning that these materials were not cytotoxic. In addition, to determine whether the three materials have any benefit in bone formation, osteogenic activities such as osteogenic differentiation and expression of differentiation marker genes were conducted in the osteoblast cell line BRITER [45]. Osteoblast differentiation was evaluated by the degree of ALP induction. All the experimental bone blocks appeared to contribute similarly to differentiation. Furthermore, under the differentiation process treated with the three substitutes, ALP, Osterix, and Osteocalcin, which are specific molecules that are expressed at each stage of differentiation, had similar levels of expression. These results show that these xenogeneic bone substitutes do not significantly affect cell proliferation and its environment and can have similar effects on bone regeneration or bone formation processes.

According to Ludwig et al. [46], re-vascularization is a prerequisite for re-proliferating bone formation cells, and cancellous bones occur faster than cortical bones, and bone reinforcement also occurs faster. Such a difference seems to derive from the multi-porous structure and pore size in cancellous bones. In the present in vivo experiment using the rabbit calvaria defect model, new bone volume and new bone area in the Cancellous and 2layer groups were significantly higher than the Cortical group with low porosity. Both cancellous bone-including groups showed uniformity of bone formation with new bones penetrating to the center of the defects. It was possible to rapidly re-vascularize due to the high porosity rate and interconnective spaces of the Cancellous and 2layer groups, and evenly distributed osteoblasts seem to influence new bone formation. Additionally, as shown in the histologic slides, newly formed bones surrounded by osteoblasts were found in the Cancellous group and the cancellous part of the 2layer group compared to the cortical bone, which indicates that cancellous bones provide appropriate space for the proliferation of bone cells and angiogenesis. Even though the compressive strengths of the Cancellous and 2layer groups were significantly lower than the Cortical, it was observed that the cancellous bone blocks served as a sufficient scaffold without structural collapse at 8 weeks after the implantation.

This in vivo study preliminarily focused on the bone regeneration efficiency of bone bocks with a two-layer structure in a critical-sized defect model. The bone scaffolds including cancellous bones, the Cancellous and 2layer groups, presented outstanding bone regeneration in the rabbit calvarial defects. In terms of producing, the bone blocks were easily trimmed without breaking or crumbling; thus, we have confirmed the potential of being able to give it proper appearances that suit the designated defect using a dental milling system. The results of the 2layer scaffold may demonstrate its sufficient efficiency in repairing a bony defect, producing enough spaces for regenerated bones. However, this study had limitations in regulating the ratio of cortical to cancellous bones in the two-layer block bones, due to the limited specimen cylindrical deign and size of 2 mm thickness. Therefore, further studies on the two-layer block bone with various bone ratios and customized bone designs should be performed in a large animal model that can reproduce the clinical situation with extensive bone loss.

## 4. Materials and Methods

### 4.1. Preparation of Porcine Bone Scaffolds

**Figure 11:**
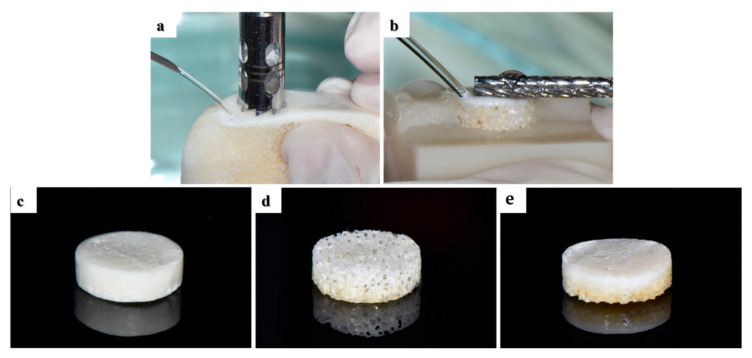
Fabrication of porcine bone blocks. (**a**) Bone block preparation using a trephine bur. (**b**) Flattening of cortical bone using a denture bar. Prepared specimens of the (**c**) Cortical, (**d**) Cancellous, and (**e**) 2layer groups.

In this study, three different porcine-derived bone scaffolds were prepared (Figure 1): cortical bone (Cortical group), cancellous bone (Cancellous group), and two-layer bone (2layer group; cortical and cancellous) composed of cortical and cancellous bone. Bones obtained from the thoracic vertebrae of porcine were submitted to high-temperature treatment for bone sintering (900 °C), and organic elements and proteins were removed by organic solvent. Cylindrical cortical, cancellous, and 2layer bone scaffolds were formed with a diameter of 6 mm using a trephine bur (outer diameter: 6 mm, 3i Implant Innovations Inc., Palm Beach Garden, FL, USA) from the porcine bone block (Figure 11a). The curved cortical bone surface of the bone blocks was flattened using a denture bur (364 CE-023, NTI-Kahla, Kahla, Germany) (Figure 11b), and they were uniformly processed to a thickness of 2 mm with a 3D-printed mold. Prepared specimens were sterilized for cell and animal experiments.

### 4.2. In Vitro Study

#### 4.2.1. Surface Observation and Chemical Composition

Surface morphologies of the Cortical and Cancellous bone blocks were observed by a scanning electron microscope (FE-SEM, Zeiss Gemini 500, ZEISS, Oberkochen, Germany). After Au coating using a sputter coater (SCD 005, BAL-TEC, Balzers, Liechtenstein), sputter-coated specimens were examined at an accelerating voltage of 10 kV at magnifications of ×50, ×300, and ×3000. Surface elemental and chemical analyses were performed by energy-dispersive X-ray spectroscopy (EDS, Oxford Link ISIS 300, Oxford, UK). EDS mapping was performed on three different areas of each specimen.

#### 4.2.2. Compressive Strength

Compressive strength of each group was measured using a universal testing machine (3366, Instron Co., Ltd., Norwood, MA, USA) at a loading of 0.5 ± 0.1 mm/min. The results are shown in a diagram as a stress (N/cm^2^, log scale) versus distance (µm, linear scale) plot. The maximum stress (N/cm^2^) was recorded after specimen fracture.

#### 4.2.3. Porosity Measurement

All block bone specimens were imaged on a micro CT system (VtomeX m 240, Baker Hughes, Houston, TX, USA) by using the following imaging parameters: voltage of 80 kV, current of 240 µA, and voxel size of 8 µm. Volume of the bone blocks was analyzed using the data processing software (VGStudio Max 3.0, Volume Graphics GmbH, Heidelberg, Germany). Porosity was calculated as the ratio of the pore volume to total volume.

#### 4.2.4. Preparation of Extracts for In Vitro Cell Assay

Next, 0.15 g of each bone block (Cortical, Cancellous, and 2layer) was mixed with 25 mL of free alpha-modification of Eagle’s medium (α-MEM; Welgene, Deagu, Korea) and stored at 37 °C and 5% CO_2_ for 1 day. Each supernatant was centrifuged once for 5 min at 1200× *g*. These supernatants were filtered through the membrane (pore size: 0.2 μm) and stored at 4 °C before use. These bone block substitute extract solutions were diluted from culture media to 20% and then treated to cells.

#### 4.2.5. Cell Cultures and Differentiation

BMP Responsive Immortalized Osteoblast Reporter cells (BRITER) were purchased from Kerafast (MA, Boston, MA, USA) and cultured in DMEM/high glucose (Hyclone, Marlborough, MA, USA) supplemented with 10% FBS in 5% CO_2_ at 37 °C for cell viability assay. Human Periodontal Ligament Fibroblasts (hPDLF) and human Gingival Fibroblasts (hGnF) were purchased from ScienCell and cultured in alpha-modification of Eagle’s medium (α-MEM) with 10% FBS in 5% CO_2_ at 37 °C. Cells were passaged after reaching 90% confluence into a 10 mm culture dish. The cells were then seeded on 48-well plates (2 × 10⁴ cells/well) and incubated for 1 day for osteogenic differentiation. After 1 day, the osteoblast differentiation was induced with 100 ng/mL of BMP2 (Cowell Medi, Busan, Korea) in the presence of growth media or diluted bone block substitute extract media for 2 and 4 days, and the media were changed regularly every 2 days.

#### 4.2.6. Cell Viability Assay

Cells were seeded on a 96-well plate at the density of BRITER 5 × 10^3^ cells/well, hGnF and hPDLF 1 × 10⁴ cells/well, and cultured for 0, 1, 2, and 3 days in a basal culture media or diluted bone block substitute extract media containing 10% FBS. After 0, 1, 2, and 3 days, cell viability was measured by a CCK-8 assay kit (Cell Counting Kit-8, Dojindo Laboratories, Kumamoto, Japan) according to the manufacturer’s protocol.

#### 4.2.7. Alkaline Phosphatase (ALP) Staining and Activity Assay

BRITER cells were seeded on a 48-well plate at the density of 2 × 10⁴ cells/well. Cells were cultured in the osteogenic media or osteogenic media containing diluted bone block substitute extracts. Each bone block substitute extract media was changed every 2 days and staining was performed on 2 and 4 days of culture. Staining used the Leukocyte Alkaline Phosphatase Kit (Sigma-Aldrich) according to the manufacturer’s protocol. Staining images were obtained using a microscope (Nikon, Eclipse Ts2, Tokyo, Japan). Quantification of the ALP-positive area images was performed using an Image J software program (U.S. National Institutes of Health, Bethesda, MD, USA).

#### 4.2.8. Quantitative Real-Time Polymerase Chain Reaction (qPCR) Analysis

BRITER cells were seeded on a 24-well plate at the density of 3 × 10⁴ cells/well. Total RNA was purified using the RNeasy mini kit (Cat#74106, Qiagen, GmBH, Hilden, Germany) according to the manufacturer’s protocol, and 2 μg of RNAs was reverse-transcribed under standard conditions with Superscript II (Invitrogen, Carlson, CA, USA). For quantitative real-time PCR analysis, 50 ng of cDNA was mixed with SYBR Green PCR Master Mix (Applied Biosystems, Forster, CA, USA) and amplified for 40 cycles in AB7500 instruments (Applied Biosystems). All samples were performed in triplicate. The data were normalized to the expression of *β-actin* mRNA and were analyzed using the 2^−∆∆Ct^ method. The primer sequences used in real-time PCR are shown in Table 4.

### 4.3. In Vivo Study

#### 4.3.1. Animals and Surgical Procedures

**Figure 12:**
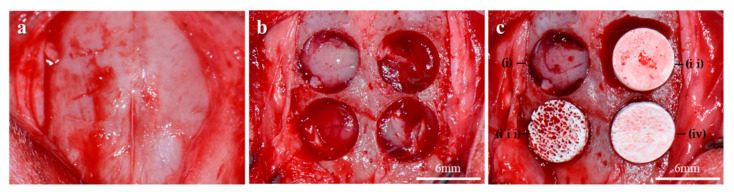
Surgical procedures using the rabbit calvaria defect model. (**a**) Exposed rabbit calvarium. (**b**) Creation of four circular bony defects. (**c**) Specimen placements in the defects. i: Control, ii: Cortical group, iii: Cancellous group, iv: 2layer group.

Six New Zealand white rabbits (12–14 weeks, mean weight: 2.5–3.5 kg) were used in this animal experiment. This study was approved by the Institutional Animal Care and Use Committee of Chonnam National University (CNU IACUC-YB-2018-94). The rabbits were sedated by being premedicated with 3 mg/kg Xylazine (Rompun^®^, Bayer Korea Ltd., Korea) via intramuscular injection. Anesthesia was induced with 10 mg/kg Tiletamine/Zolazepam (Zoletil^®^, Virbac Korea, Korea) intramuscularly and maintained with 1–2% isoflurane (Ifran Liq, Hana Pharm, Korea). The surgical site of the rabbit cranium was shaved and disinfected with betadine and the site was locally injected with 2% lidocaine (1:100,000 epinephrine, Yu-Han Co., Gunpo, Korea). A 2 cm sagittal incision along the midline of the calvarium was made using a #15 surgical blade, and full-thickness flaps were raised (Figure 12a). After removal of the periosteum, four circular calvaria defects (6 mm in a diameter) were formed on each rabbit using a trephine bur (3i Implant Innovations Inc., Palm Beach Garden, FL, USA) under continuous saline irrigation (Figure 12b). Bone scaffolds of each group were randomly grafted in the calvaria defects (Figure 12c), for the negative control; no graft material was assigned to the defect. Afterward, the incised skin was sutured using a 4-0 absorbable suture (Vicryl, Ethicon, Somerville, NJ, USA). Eight weeks after the surgical experiment, experimental animals were euthanized under CO_2_ gas. Calvarias were harvested and then immersed in 10% formalin for 7 days.

#### 4.3.2. Micro-Computed Tomography (µCT) Analysis

**Figure 13:**
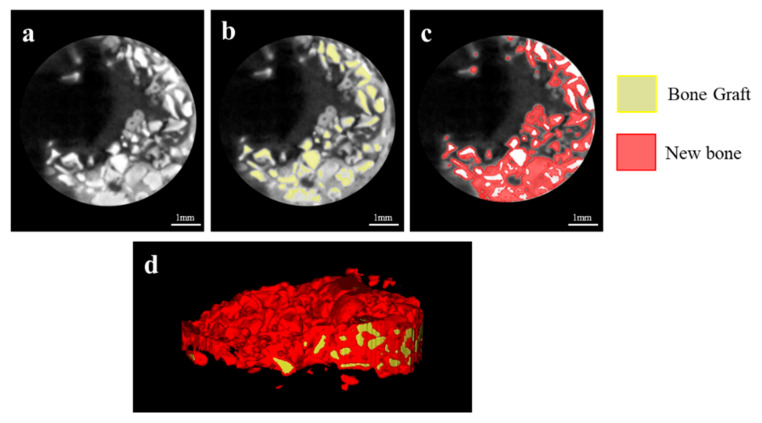
Images within ROIs obtained by micro-computed tomography analysis. (**a**) Two-dimensional micro CT image; colored images of (**b**) grafted bone scaffold and (**c**) new bones. (**d**) Converted 3D image for adjudging external appearance.

µCT three-dimensional images were obtained to determine the new bone volume at the defect site by using a µCT imaging system (Quantum GX, PerkinElmer, Hopkinton, MA, USA). Considering the scaffold included in the calvaria tissue, the image quality was maintained and measurable by applying a voltage of 90 kV, pixel spacing of 80 µm, and intensity of 160 mA. The three-dimensional (3D) volumes of the defect and scaffold were measured by MeshLab software (Mathworks, Natick, MA, USA). The 3D model of the bone scaffold obtained by image segmentation was converted into STL format to accurately adjudge the external form of a 3D model. The converted file was imported from 3D-processing software (Blender Foundation, Blender^TM^, Amsterdam, Netherlands) and rendered. The region of interest was adjusted to a diameter of 6 mm (Figure 13) and height of 2 mm, and new bone volume (NBV; MM^3^) within the region of interest was calculated.

#### 4.3.3. Histomorphometric Analysis

**Figure 14:**
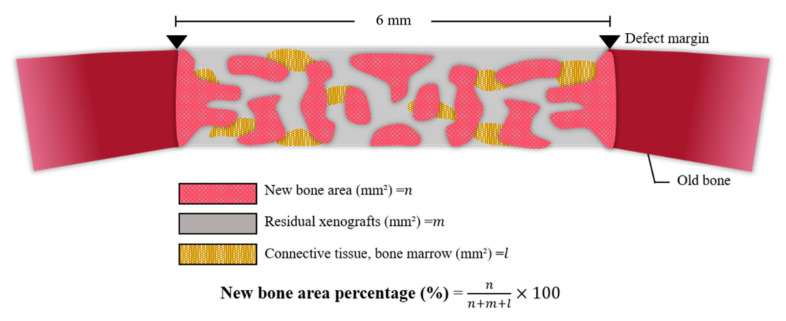
Schematic diagram for histometric analysis.

Formalin fixed tissues were decalcified using Calci-Clear Rapid (National Diagnostics, 305 Patton Drive Atlanta, GA 30336, USA). After the dehydration procedure, the tissues were embedded in paraffin and were sectioned longitudinally at 4 µm using a fully automated rotary microtome (Leica RM2255, Leica Microsystems, IL, USA). Tissue slides were stained with hematoxylin eosin (H&E) and Masson’s trichrome (MT) stains to visualize newly regenerated bones for the histomorphometric analysis. Images of slides were captured using a microscope (Olympus BX, Tokyo, Japan) equipped with a CCD camera (Polaroid DMC2 digital Microscope Camera, Polaroid) and were evaluated using an i-Solution image program (IMT, Daejeon, Korea) by a single experimental expert. The results are expressed as mean and standard deviation of new bone area (%) (Figure 14).

#### 4.3.4. Statistical Analysis

The statistical analysis was undertaken using statistical software (SPSS ver. 25.0, Chicago, IL, USA). The in vitro results were analyzed by one-way ANOVA followed by Bonferroni’s post-hoc U test. In the in vivo study, the Kruskal–Wallis test followed by the Mann–Whitney U post-hoc test was performed to compare the results of new bone volume; the one-way ANOVA followed by the Bonferroni post-hoc test was carried out for the histometric result. The significance of differences was accepted for *p* value < 0.05.

## 5. Conclusions

Within the limitations of this in vivo study, the cancellous and two-layer porcine-derived bone scaffolds showed satisfactory bone regeneration efficiency; further studies on regulating the ratio of the cortical and cancellous bones are needed.

## Figures and Tables

**Table 1:** Energy-dispersive X-ray spectroscopy (EDS) (%).

Elements	Chemical Compositions (wt %)	
	Cortical	Cancellous
C	13.79 ± 9.32	5.63 ± 0.26
O	41.37 ± 4.07	38.63 ± 4.46
Na	0.77 ± 0.65	0.29 ± 0.16
Mg	0.20 ± 0.20	0.37 ± 0.12
P	14.78 ± 3.59	17.72 ± 1.27
Ca	29.04 ± 9.29	37.35 ± 4.05
Ca/P	1.96	2.11

**Table 2:** Mean and standard deviation (SD) of new bone volume (*n* = 6).

	Groups	Mean	SD	*p*-Value
New bone volume (%)	Control	4.58	1.06	0.001 ***
	Cortical	14.45	3.72	
	Cancellous	37.02	8.20	
	2layer	27.67	3.50	

*** *p* < 0.001.

**Table 3:** Mean and standard deviation (SD) of new bone area (*n* = 6).

	Groups	Mean	SD	*p*-Value
New bone area (%)	Control	8.37	3.77	0.019 *
	Cortical	13.62	5.86	
	Cancellous	37.76	7.44	
	2layer	29.12	6.61	

* *p* < 0.05.

**Table 4:** List of primer sequences used for polymerase chain reaction (PCR) analysis.

Target Genes	Sequences
*β-actin*	F: 5′-TCTGGCACCACACCTTCTAC-3′
	R: 5′ -TACGACCAGAGGCATACAGG- 3′
*ALP*	F: 5′- TGACCTTCTCTCCTCCATCC- 3′
	R: 5′-CTTCCTGGGAGTCTCATCCT-3′
*Osteocalcin*	F: 5′- GCAATAAGGTAGTGAACAGACTCC -3′
	R: 5′ -GTTTGTAGGCGGTCTTCAAGC- 3′

## Data Availability

The data presented in this study are available on request from the corresponding author.
